# Ivabradine Induces Cardiac Protection against Myocardial Infarction by Preventing Cyclophilin-A Secretion in Pigs under Coronary Ischemia/Reperfusion

**DOI:** 10.3390/ijms22062902

**Published:** 2021-03-12

**Authors:** Ignacio Hernandez, Laura Tesoro, Rafael Ramirez-Carracedo, Javier Diez-Mata, Sandra Sanchez, Marta Saura, Jose Luis Zamorano, Carlos Zaragoza, Laura Botana

**Affiliations:** 1 Hospital Ramón y Cajal Research Unit (IRYCIS), Cardiology Department, Universidad Francisco de Vitoria, 28223 Madrid, Spain; naxete1992@gmail.com (I.H.); lauratesoro4@hotmail.com (L.T.); rrcarracedo@hotmail.com (R.R.-C.); jdiezmata@gmail.com (J.D.-M.); laura.botana@ufv.es (L.B.); 2 CIBER de Enfermedades Cardiovasculares (CIBERCV), Instituto de Salud Carlos III (ISCIII), 28029 Madrid, Spain; marta.saura@uah.es (M.S.); zamorano@secardiologia.es (J.L.Z.); 3 Systems Biology Department, Universidad de Alcala, IRYCIS, 28772 Alcala de Henares, Spain; sandrasanchez07@hotmail.com; 4 Cardiology Department, Hospital Ramón y Cajal, IRYCIS, 28034 Madrid, Spain

**Keywords:** acute myocardial infarction, ischemia/reperfusion, Ivabradine, EMMPRIN, cyclophilin A

## Abstract

In response to cardiac ischemia/reperfusion, proteolysis mediated by extracellular matrix metalloproteinase inducer (EMMPRIN) and its secreted ligand cyclophilin-A (CyPA) significantly contributes to cardiac injury and necrosis. Here, we aimed to investigate if, in addition to the effect on the funny current (I(f)), Ivabradine may also play a role against cardiac necrosis by reducing EMMPRIN/CyPA-mediated cardiac inflammation. In a porcine model of cardiac ischemia/reperfusion (IR), we found that administration of 0.3 mg/kg Ivabradine significantly improved cardiac function and reduced cardiac necrosis by day 7 after IR, detecting a significant increase in cardiac CyPA in the necrotic compared to the risk areas, which was inversely correlated with the levels of circulating CyPA detected in plasma samples from the same subjects. In testing whether Ivabradine may regulate the levels of CyPA, no changes in tissue CyPA were found in healthy pigs treated with 0.3 mg/kg Ivabradine, but interestingly, when analyzing the complex EMMPRIN/CyPA, rather high glycosylated EMMPRIN, which is required for EMMPRIN-mediated matrix metalloproteinase (MMP) activation and increased CyPA bonding to low-glycosylated forms of EMMPRIN were detected by day 7 after IR in pigs treated with Ivabradine. To study the mechanism by which Ivabradine may prevent secretion of CyPA, we first found that Ivabradine was time-dependent in inhibiting co-localization of CyPA with the granule exocytosis marker vesicle-associated membrane protein 1 (VAMP1). However, Ivabradine had no effect on mRNA expression nor in the proteasome and lysosome degradation of CyPA. In conclusion, our results point toward CyPA, its ligand EMMPRIN, and the complex CyPA/EMMPRIN as important targets of Ivabradine in cardiac protection against IR.

## 1. Introduction

Cardiac remodeling is the result of an orchestrated series of genomic, biochemical, and morphological changes which determine the heart’s fate in terms of restoring adequate heart contractility. Adverse cardiac remodeling may result in severe and chronic heart failure by still unknown molecular events, and hence, intense research on finding new diagnostic and forecast molecular targets is of a significant interest.

Ivabradine is designed to target cardiac funny current (I(f)) by inhibiting the activity of hyperpolarization-activated cyclic nucleotide (HCN)-gated potassium channels, reducing heart rate with no significant effect on blood pressure and cardiac output. Therefore, Ivabradine is efficiently administered for the management of acute heart failure [1]. We and others have confirmed that Ivabradine is also effective against dobutamine-induced tachycardia in a cardiac shock (CS) model, improves the hemodynamic parameters immediately after acute myocardial infarction (AMI) and in the long term [2,3], and inhibits extracellular matrix metalloproteinase inducer (EMMPRIN) activity in a porcine model of coronary ischemia/reperfusion (IR), but the underlying molecular pathways are yet to be understood.

Cyclophilins are key inflammatory mediators in the context of several pathologies by at least serving as natural ligands of EMMPRIN. Disruption of the complex EMMPRIN/cyclophilin-A was effective in reducing infarct size by preserving the systolic function in mice subjected to coronary IR [4].

We reported the contribution of EMMPRIN to the onset and progression of cardiovascular diseases in mice, including AMI and atherosclerosis [5,6,7] and in porcine models of IR [8]. We recently found that Ivabradine induces cardiac protection against coronary IR beyond the current I(f) through the inhibition of EMMPRIN [3]. Therefore, here, we hypothesize that the better outcomes experienced by patients in response to Ivabradine could be attributed, at least in part, to preventing cyclophilin-A release and/or binding to high-glycosylated EMMPRIN in the heart.

## 2. Results

### 2.1. Ivabradine Induces Cardiac Protection of Pigs against IR

**Figure 1:**
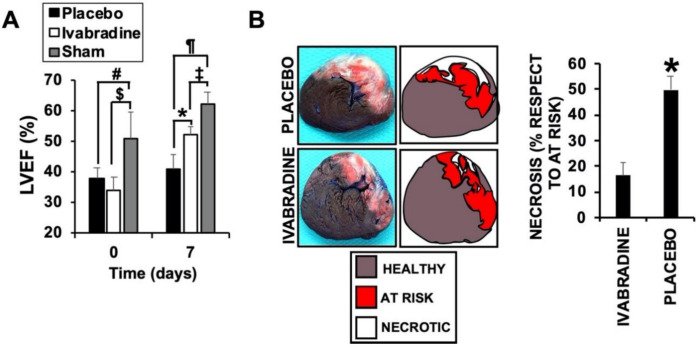
Ivabradine induces cardiac protection in pigs subjected to ischemia/reperfusion (IR). (**A**) Left ventricle ejection fraction values obtained at time points 0 and 7 days post-IR (*n* = 9 Ivabradine/8 placebo/3 sham. Mean ± SD. # *p* < 0.001 day 0 placebo vs. sham. $ *p* < 0.001 day 0 Ivabradine vs. sham. * *p* < 0.05 day 7 placebo vs. Ivabradine. ¶ *p* < 0.001 day 7 placebo vs. sham. ‡ *p* < 0.001 day 7 Ivabradine vs. sham. (**B**) Evans blue/TTC double staining performed in 0.5 cm heart sections isolated 7 days after IR from pigs treated with 0.3 mg/kg Ivabradine or placebo.

In pigs injected with 0.3 mg/kg Ivabradine, a cardiac ultrasound was used to find that the left ventricle ejection fraction (LVEF) was significantly improved, when compared to a placebo, by day 7 after IR (Figure 1A) as a result of a reduction in the necrotic area (showing similar area at risk sections: placebo, 34.22% ± 8.8; Ivabradine, 29.76 ± 7.4). This was detected using Evans blue/2,3,5-Triphenyltetrazolium chloride (TTC) staining in sections of the same hearts (Figure 1B), demonstrating a cardioprotective effect of Ivabradine against IR. LVEF was also calculated in animals subjected to a sham procedure (see methods for details).

### 2.2. Ivabradine Increases the Expression of Cyclophilin-A in the Hearts of Pigs after IR

**Figure 2:**
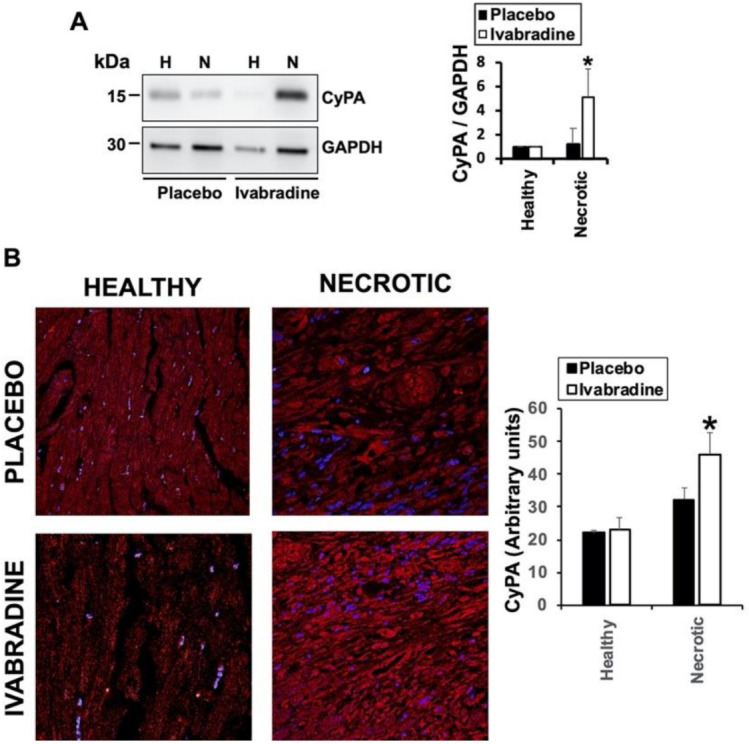
Ivabradine increases de-expression of cyclophilin-A in the hearts of pigs subjected to IR. (**A**) Immunoblot detection of cyclophilin-A (CypA) and Glyceraldehyde-3-phosphate dehydrogenase (GAPDH) as loading control in heart lysates from placebo and Ivabradine pigs subjected to IR. *n* = 9 Ivabradine/8 placebo. Mean ± SD; * *p* < 0.05 necrotic Ivabradine vs. placebo. (**B**) Confocal microscopy detection of cyclophilin-A (red) in heart sections of pigs treated with Ivabradine or placebo. *n* = 9 Ivabradine/8 placebo. Mean ± SD; * *p* < 0.05 necrotic Ivabradine vs. placebo.

We previously found that as part of the inflammatory response, myocardial infarction induces the expression of EMMPRIN in the heart, while Ivabradine, by means of still unknown mechanisms, reduced the levels of EMMPRIN after IR [7]. To test whether Ivabradine may reduce IR-induced inflammation, the expression of cyclophilin-A (CyPA), which binds to EMMPRIN and plays a key role in inflammation, was evaluated. Immunoblot detection of CyPA from protein lysates collected from healthy and necrotic areas of hearts subjected to IR showed that in response to 0.3 mg/kg Ivabradine, the levels of CyPA were increased in the necrotic areas by day 7 after IR, while in the placebo group, we found the opposite (Figure 2A). The same result was obtained by confocal microscopy detection of CyPA from the same hearts (Figure 2B).

### 2.3. Ivabradine Reduces Plasma CyPA Levels after IR

**Figure 3:**
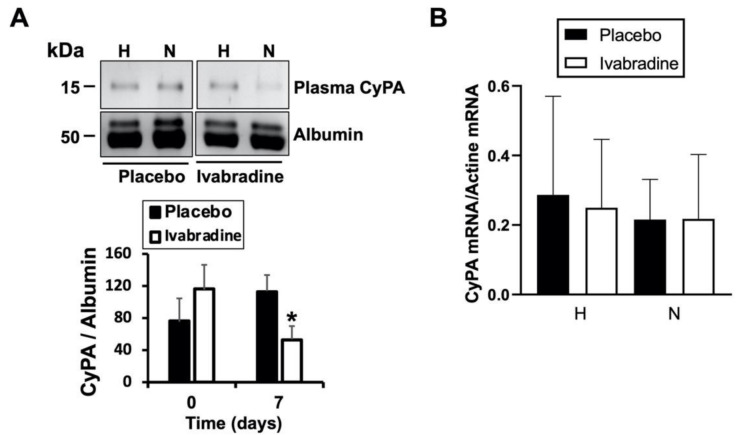
Ivabradine reduces plasma CyPA after IR. (**A**) Immunoblot detection of CyPA and albumin as loading control in plasma samples from placebo- and Ivabradinetreated pigs subjected to IR (*n* = 9 Ivabradine/8 placebo. Mean ± SD; * *p* < 0.05 d7 placebo vs. Ivabradine). (**B**) mRNA expression from healthy and necrotic sections of hearts subjected to IR in response to Ivabradine or placebo (*n* = 9 Ivabradine/8 placebo).

CyPA is secreted into the extracellular space and binds to different ligands, including EMMPRIN, triggering an inflammatory response. Here, we found that in response to 0.3 mg/kg Ivabradine, plasma levels of CyPA were significantly reduced when compared to those found in the placebo group (Figure 3A). To investigate whether Ivabradine may also regulate CyPA transcriptional expression, total RNA was isolated from the same hearts as above and did not show differences in the levels of CyPA mRNA (Figure 3B). Taken together, our data suggest that although we cannot exclude a negative effect from endogenous CyPA accumulation, Ivabradine induces cardiac protection, at least in part, by preventing the secretion of CyPA in response to IR.

### 2.4. Ivabradine Increases the Binding between CyPA and Low-Glycosylated forms of EMMPRIN

**Figure 4:**
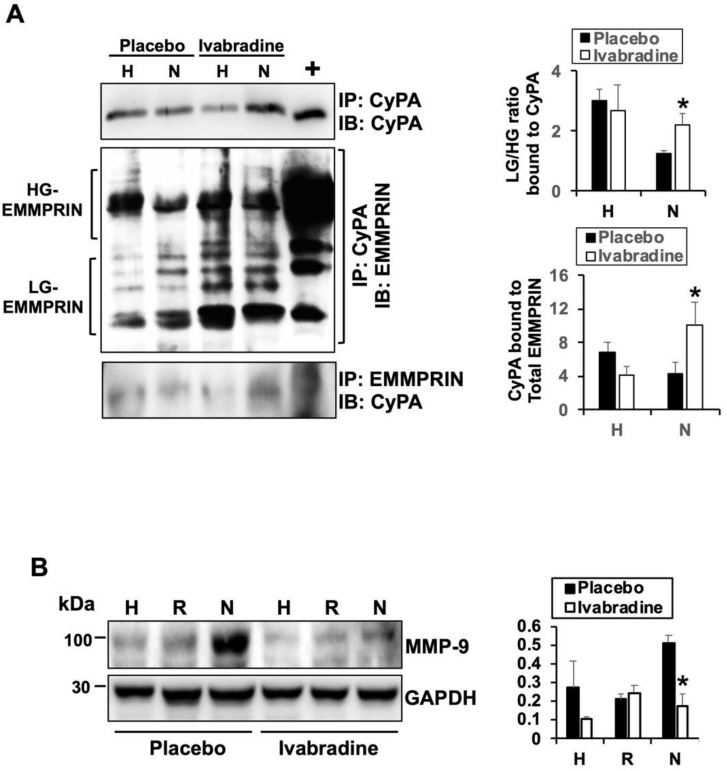
Ivabradine increases the binding between CyPA and low-glycosylated extracellular matrix metalloproteinase inducer (EMMPRIN). (**A**) Immunoprecipitation of CyPA and EMMPRIN in heart extracts (*n* = 9 Ivabradine/8 placebo, mean ± SD, * *p* < 0.05 necrotic placebo vs. Ivabradine). (**B**) Immunoblot detection of MMP-9 and GAPDH as a loading control in heart lysates from placebo- and Ivabradine-treated pigs subjected to IR (*n* = 9 Ivabradine/8 placebo. Mean ± SD, * *p* < 0.05 necrotic placebo vs. Ivabradine).

As part of the inflammatory response, high-glycosylated EMMPRIN induces the secretion and activation of several extracellular matrix-degrading enzymes by forming oligomers at the plasma membrane [9]. Hence, we analyzed the contribution of Ivabradine in the complex between CyPA and EMMPRIN in response to IR and how glycosylation of EMMPRIN might be affected. By using anti-EMMPRIN- and anti-CyPA specific antibodies, we crossed a co-immunoprecipitated protein extract isolated 7 days after IR from the hearts of pigs injected with 0.3 mg/kg Ivabradine or placebo. We found that in response to Ivabradine, more CyPA preferentially binds to low-glycosylated EMMPRIN (LG-EMMPRIN, Figure 4A) when compared to a placebo. Subsequently, the expression of the downstream MMP-9 was significantly reduced in response to ivabradine, as we previously detected (Figure 4B, approved to reproduce Figure 4B by Elsevier “Revista Española de Cardiología” (Engl Ed). 2020 Oct 29:S1885-5857(20)30415-1. doi: 10.1016/j.rec.2020.09.012). Taken together, our data show that beyond the current I(f), Ivabradine may induce cardiac protection against myocardial infarction by preventing the secretion of CyPA and binding to LG-EMMPRIN to prevent ECM-degrading MMP-9 expression in response to IR.

### 2.5. Ivabradine Reduces the Secretion of CyPA in Cardiac Cells

**Figure 5:**
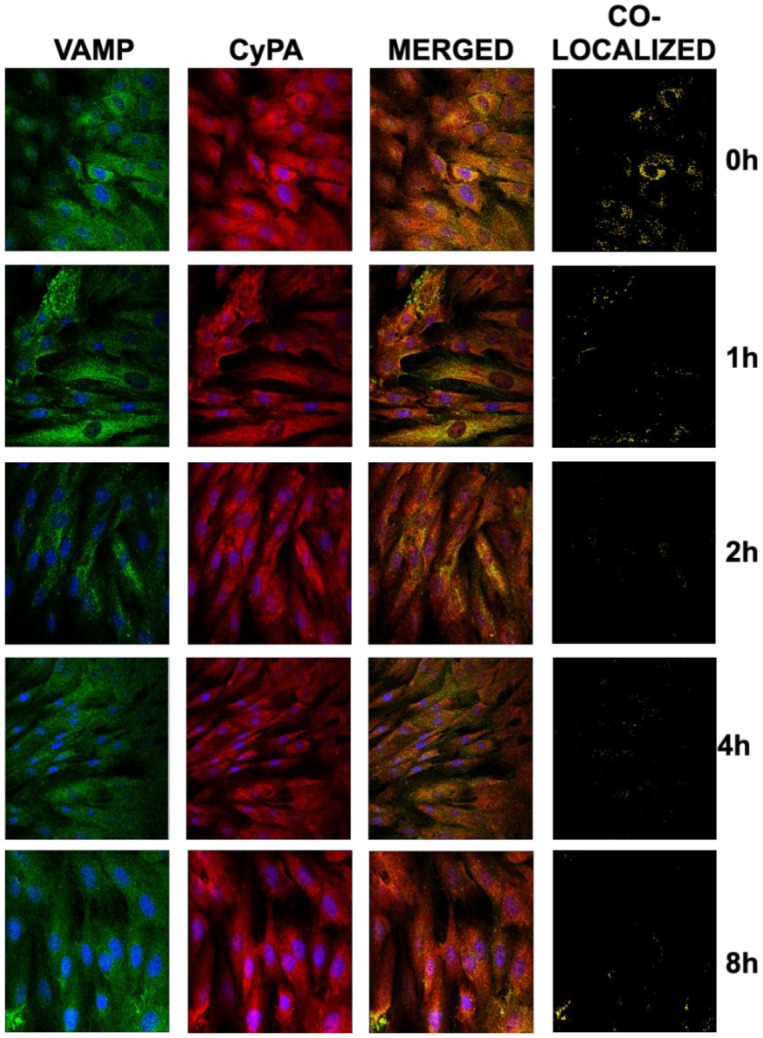
Ivabradine reduces the secretion of CyPA in cardiac cells. H9c2 cells were treated with 5 mM Ivabradine for 0,1,2,4, and 8 h. CyPA (red) and VAMP (green) were visualized with specific antibodies by confocal microscopy. Merged panels show co-localization in yellow. Co-localized panels were generated through analyzing co-localizing pixels with the ImageJ software package. Nuclei were stained with dye Hoechst (*n* = 3).

Others have demonstrated that CyPA secretion requires vesicle transport, docking, and fusion to the plasma membrane, although the factors that regulate CyPA granule release are not fully explored. To test whether Ivabradine may also have an impact on the granule secretion of CyPA, we incubated cardiac H9c2 cells with Ivabradine and visualized the location of CyPA and the soluble N-ethylmaleimide-sensitive factor (NSF) attachment protein receptor (SNARE) VAMP-1, a component of the exocytosis machinery present in cardiac myocytes [10], by confocal microscopy. We found that CyPA and VAMP-1 co-localize in cardiac cells, indicative of granule secretion of CyPA (Figure 5 upper panels, t0). By contrast, co-localization of both proteins was significantly reduced in the first hour of incubation with Ivabradine in a time-dependent manner (Figure 5).

### 2.6. Ivabradine Decreases Lysosomal Degradation of CyPA in Cardiac Cells

**Figure 6:**
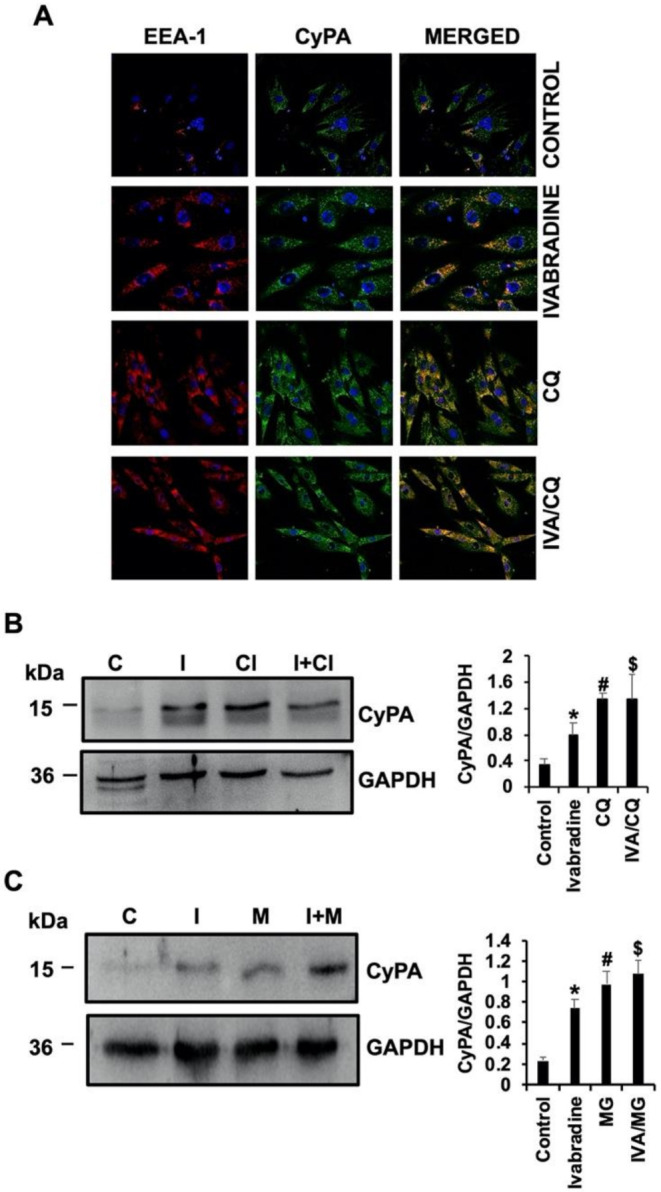
Ivabradine has no effect on CyPA degradation. (**A**) Confocal microscopic detection of EEA-1 (red) and CyPA (green) in H9c2 cells incubated with Ivabradine, chloroquine, and a combination of both. Merged panels show co-localization in yellow (*n* = 3). (**B**) Immunoblot detection of CyPA and GAPDH as a loading control in protein lysates from cells incubated with Ivabradine, chloroquine, and a combination of both (*n* = 3, mean ± SD, * *p* < 0.03 control vs. Ivabradine. ^#^ *p* < 0.01 control vs. chloroquine (CQ), ^$^ *p* < 0.006 control vs. CQ/iva). (**C**) Immunoblot detection of CyPA and GAPDH in protein lysates from cells incubated with Ivabradine, MG-132, and a combination of both (*n* = 3. Mean ± SD; * *p* < 0.003 control vs. Ivabradine. ^#^ *p* < 0.005 control vs. MG. ^$^ *p* < 0.005 control vs. MG/iva).

We found that Ivabradine increases the levels of CyPA in the hearts of pigs, by preventing its secretion in response to IR, but such an accumulation could also result from a defective CyPA proteolytical degradation. To test this hypothesis, we first investigated the lysosomal degradation of CyPA in the presence of 5 mM Ivabradine and 10 µM of the lysosomal inhibitor chloroquine (CQ) in H9c2 cells. Confocal microscopic detection of CyPA with the early endosome marker early endosome-associated protein (EEA1) showed co-localization of both proteins in response to Ivabradine, CQ, and the combination of both stimuli, suggesting that early endosomal accumulation of CyPA may respond to the inhibition of a downstream lysosomal activity (Figure 6A). However, CyPA was accumulated in the presence of CQ, as detected by immunoblot, while co-incubation with Ivabradine was not affected, suggesting that Ivabradine has no effect on the lysosomal-induced degradation of CyPA (Figure 6B). The same was found by culturing H9c2 cells under hypoxic conditions (Supplementary Figure S1). In addition, the incubation of cardiac H9c2 cells with 5 mM of Ivabradine and 15 µM of the proteasome pharmacological inhibitor MG-132 had the same effect, while co-incubation with both substances had no synergic effect on CyPA concentration (Figure 6C).

## 3. Discussion

In the current work, we show an underlying mechanism to prevent the inflammatory response elicited by cardiac IR in response to Ivabradine. Ivabradine prevents the granule secretion of CyPA and increases lysosomal-induced CyPA degradation, thus preventing binding to its receptor for extracellular matrix metalloproteinase inducer (EMMPRIN), as a mechanism to avoid extracellular matrix degradation.

CyPA is an intracellular protein that is expressed under inflammatory conditions by several cell types; some of them have been shown to secrete CyPA during in the early stages of pathologies that include atherosclerosis, coronary artery disease (CAD), and acute myocardial infarction [11]. During atherosclerosis, CyPA mediates ox-LDL-induced macrophage activation [12], and it was recently shown that inhibition of CyPA degradation is also prevented by ox-LDL via a mechanism which enlarges early atherosclerotic burden [13]. In addition to macrophages, platelets also bind to CyPA in patients with significant coronary artery disease and are correlated with hypercholesterolemia and hypertension [14]. Here, we found that Ivabradine not only prevents the secretion of CyPA but also induces its lysosomal-mediated degradation.

We have shown that Ivabradine decreases the secretion of CyPA by a vesicular pathway observing a lower compartmentalization of CyPA in vesicles labeled with VAMP-1. Previous studies have indicated that reactive oxygen species (ROS)-induced CyPA secretion from vascular smooth muscle cells (VSMC) requires a highly regulated process of vesicle transport, docking, and fusion at the plasma membrane, with VAMP-1 being one of the major proteins in this pathway [15]

In recent years, the role of CyPA in coronary artery disease has been investigated. As recently reported, plasma CyPA is a biomarker of CAD [16], showing that a progressive decrease in plasma CyPA predicts better ventricular performance in patients with STEMI [17]. After coronary reperfusion, super-oxygenation triggers an inflammatory response which leads to an excessive production of reactive oxygen species (ROS). Cardiac remodeling is the underlying response which at the end induces cardiac hypertrophy leading to acute heart failure. Excessive levels of ROS stimulate the secretion of CyPA which at the end contributes to increasing the production of ROS [15] by still unknown mechanisms. Nevertheless, and although chemokines are the key components in inflammatory cell trafficking, recent data show that CyPA is also a chemical attractor for leukocytes [18]. Here, we found that Ivabradine prevents plasma secretion of CyPA, suggesting a new anti-inflammatory mechanism through an inhibition of leukocyte recruitment to the lesion.

Intracellular CyPA may play a role in the pathogenesis of cardiovascular diseases by acting as a chaperone and playing a role in protein folding, trafficking, and function [19]. However, recent findings show that a significant pool of CyPA is an intra-mitochondrial factor, enhancing antiapoptotic signals, and hence suggesting a role in cell survival [20]. Indeed, CyPA was found to inhibit oxidative stress and apoptosis by modulating the PI3K/Akt/mTOR signaling pathway [21] in cancer cells. Here, we found that in response to Ivabradine, CyPA was accumulated in the necrotic area of pigs subjected to IR when compared to the placebo group.

In the cardiovascular system, and as we mentioned above, a progressive decrease in plasma CyPA may forecast better ventricular performance in patients with STEMI [17]. In the current work, we found that Ivabradine, rather than inducing CyPA expression, inhibited CyPA secretion in cardiac cells, which may contribute to explain the differences between extracellular and intracellular CyPA in the placebo vs. Ivabradine groups. Therefore, and although we cannot exclude any negative effect from accumulating intracellular CyPA, we may suggest that Ivabradine induces cardiac protection, by at least preventing the secretion of CyPA, which has been previously reported as mechanism involved in cardiovascular disease. Bayon and co-authors have recently shown that plasma levels of CyPA correlated with the occurrence of coronary artery disease, when compared to a control group of healthy patients [22]. Here, we also found that pigs with reduced plasma levels of CyPA had better cardiac function after IR.

EMMPRIN has been identified as a major receptor of CyPA on leukocyte signaling [23]. We and others found that inhibition of EMMPRIN by means of different strategies promotes cardiac protection in humans [24] and in animals subjected to coronary ischemia/reperfusion, including incubation with anti-EMMPRIN-specific antibodies [5] and EMMPRIN-targeted magnetic nanoparticles [6] in mouse and pig models of IR. The complex CyPA/EMMPRIN worsens myocardial damage by contributing to leukocyte recruitment [4,25] and also by inducing extracellular matrix degradation through EMMPRIN-induced matrix metalloproteinase activation [26]. Recently, we found that Ivabradine induces cardiac protection by inhibiting EMMPRIN-mediated MMP activation [8,27], but the specific role of the complex CyPA/EMMPRIN has not been previously investigated.

Lately, SP-8365—a new inhibitor of the complex CyPA/EMMPRIN—was found effective for the treatment of atherosclerosis by reducing plaque progression and stabilizing vulnerable plaque in apoE-deficient mice [28]. Here, we found that rather than disrupting the complex, Ivabradine increased the binding between CyPA and LG-EMMPRIN, a low-molecular glycosylation form of EMMPRIN, which was not associated with EMMPRIN-induced MMP expression and activation [29].

In conclusion, we propose the use of Ivabradine as a mechanism to prevent the inflammatory signaling elicited in response to acute myocardial infarction by inhibiting the secretion and binding of CyPA to its receptor EMMPRIN, as a way to avoid extracellular matrix degradation and necrosis. Beyond its use in acute and chronic heart failure patients, Ivabradine may be effective in other inflammatory cardiovascular diseases such as atherosclerosis, as others recently found a significant improvement by early administration of Ivabradine in apoE-null mice [30]. Additionally, the use of Ivabradine in inflammatory non-cardiovascular diseases may also represent a new window of research for the treatment of other inflammatory pathologies.

## 4. Materials and Methods

### 4.1. Reagents

Hematoxylin-eosin, trichrome Masson staining reagents, TTC, Evans blue, MG132, chloroquine, and fetal bovine serum were from Sigma (Saint Louis, MO, USA). Horse radish peroxidase (HRP)-conjugated anti-mouse secondary antibody and liquid 3,3’-diaminobenzidine (DAB) substrate were from Dako (Santa Clara, CA, USA). Anti-MMP-9 and anti-CyPA antibodies were from Abcam (Cambridge, UK), ketamine was from Pfizer (New York, NY, USA), isoflurane was from Abbvie (North Chicago, IL, USA), propofol was from Fresenius (Bad Homburg, Germany), fentanyl was from Kern Pharma (Madrid, Spain), diazepam was from Roche (Basel, Switzerland), and amiodarone was from Sanofi Aventis (Gentilly, France).

### 4.2. Cardiac Ischemia/Reperfusion

Animal procedures were performed in the Experimental Surgery Department of the Hospital Universitario La Paz (Madrid, Spain). The investigation conforms to the Guide for the Care and Use of Laboratory Animals published by the US National Institutes of Health (NIH Publication No. 85–23, revised 1985) and the Animal Welfare Ethics Committee and complied with the EU Directive on experimental animals (63/2010 EU) and related Spanish legislation (RD 53/2013). PROEX 365-15 is the procedure approved (10 January 2020) by the local government ethics committee in accordance with the practical guidelines for rigor and reproducibility in preclinical and clinical studies on cardioprotection [31,32].

Before experimentation, animals were housed for one week to avoid cardiovascular responses derived from anxiety associated with the new environment. Prior to experimentation, the animals underwent a cardiac ultrasound to check for abnormalities in cardiac anatomy and function.

Yorkshire female pigs (38.9 ± 2.99 kg) were pre-medicated with intramuscular ketamine 10 mg/kg (Pfizer) and midazolam 0.5 mg/kg (B. Braun). Anesthesia was induced by inhaled isoflurane (Abbvie) and maintained with a continuous infusion of propofol 2 mL/kg/h (Fresenius), fentanyl 50 µg/kg/h (Kern Pharma), and diazepam 10 µg/kg/h (Roche). Animals were intubated and ventilated with 100% oxygen saturation. The animals received 5000 IU of heparin and amiodarone 2 mg/kg/h (Sanofi Aventis) to avoid blood clotting in catheters and malignant cardiac arrhythmias, respectively. Before balloon inflation, their coronary anatomy was visualized to check for vasculature abnormalities.

Ischemia/reperfusion was induced by LAD occlusion for 45 min, using a JL 3 6F catheter and an angioplasty balloon (inflated to the pressure of 8 atmospheres). In cases where ventricular fibrillation/ventricular tachycardia occurred, we administered a biphasic DC shock (10–20 joules) combined with direct manual chest compressions. After 45 min, the balloon was deflated and the animals were randomized to placebo (saline) or 0.3 mg/kg Ivabradine. In total, 10 mL of arterial blood was obtained from the femoral artery at regular times, and the plasma was isolated by centrifugation at 3000 rpm for 10 min.

### 4.3. Echocardiography

Pig hearts subjected to IR or a sham procedure (animals subjected to the same procedure except balloon inflation) were visualized by echocardiography using a Vivid Q ultrasound system from GE healthcare (Chicago, IL, USA) equipped with a 1.9–4 MHz scan head. In the anesthetized animals, the parasternal short-axis view images of the heart were recorded in a B-mode to allow M-mode recordings by positioning the cursor in the parasternal short-axis view perpendicular to the interventricular septum and posterior wall of the left ventricle. From these recordings, the following parameters were determined using the on-site software cardiac package: systolic and diastolic interventricular septum thickness (IVS), systolic and diastolic left ventricle internal diameter (LVID), systolic and diastolic left ventricle posterior wall thickness (LVPW), left ventricle ejection fraction (EF), left ventricle shortening fraction (FS), heart rate (HR), and cardiac output (CO). In addition, the LVEF was also measured using the B-mode Simpson biplane method using 4-chamber and 2-chamber left ventricle long axis views, with similar results. To avoid interobserver-derived biases, data acquisition and analysis were performed by one single operator.

### 4.4. Evans Blue/TTC Staining

The extension of myocardial infarction was evaluated by Evans blue perfusion and TTC staining. By day 7, a catheter was inflated at the same position as in day 0 to avoid Evans blue perfusion downstream to the area at risk, and a pigtail catheter was inserted from the femoral artery and placed up to the left ventricle for Evans blue perfusion into the systemic circulation. One minute after perfusion, the animals were sacrificed by injection of a potassium chloride solution, and their hearts were then isolated, washed 3 times with saline buffer, frozen for 12 h at −20 °C, and chopped into 0.5 cm slices from base to apex. The slices were incubated with 1% TTC dye dissolved in a saline buffer for 20 min at 37 °C and then washed for 20 min with 10% paraformaldehyde. Images were acquired with the ImageJ software discriminating between healthy areas (blue), the area at risk (dark red), and the pale necrotic area (white), calculating the area of necrosis as percentage with respect to the area at risk.

### 4.5. RNA Isolation and RT-PCR

Total RNA was isolated from animals’ hearts using the RNeasy Mini kit according to manufacturer’s instructions (Qiagen) and quantified by spectrophotometry (NanoDrop). RNA was reverse transcribed, and the corresponding cDNAs were used as templates in PCR assays with specific PCR CyPA primers. The amplification was performed with 30 cycles consisting of denaturation for 30 s at 95 °C, primer annealing for 30 s at 66 °C, and primer extension for 60 s at 72 °C. The cDNA products were electrophoresed in 4% agarose and visualized with RedSafe staining (EcoGen).

### 4.6. Confocal Microscopy

Paraffin-embedded 5 µm heart sections were incubated with the corresponding primary antibody (diluted 1:500 in Phosphate-Buffered Saline (PBS), 1.5% Bovine serum albumin (BSA)) overnight at 4 °C. After washing 3 times with PBS, the slides were incubated with the secondary conjugated antibody for 1 h at room temperature. Slides were washed 3 times with PBS and mounted in PBS media containing Hoechst for nuclei visualization. Images were taken using a Leica TCS SP5 confocal microscope. At least three different fields per condition were obtained.

### 4.7. H9c2 Cell Culture and Treatment

H9c2 cells were grown in Dulbecco’s modified Eagle’s medium (DMEM) supplemented with 10% fetal bovine serum (FBS) from Sigma Aldrich (Saint Louis, MO, USA), 50 mg/mL of penicillin and 50 mg/mL streptomycin (Invitrogen, Waltham, MA, USA), and incubated at 37 °C in a humidified atmosphere of 5% CO_2_ and 95% oxygen. In addition, the cells were also cultured under hypoxic conditions in hypoxia incubator chambers containing a 1% oxygen, 5% CO_2_, and 94% N_2_ humidified atmosphere. H9c2 cells were treated with 5 mM Ivabradine, 15 µM MG132, 10 µM chloroquine, and the combinations at the times indicated below.

### 4.8. Protein Expression Determination by Immunoblot

Immunoblot was performed as described [5].

### 4.9. Statistical Analysis

All data were analyzed in a statistical software package (SPSS 22.0, SPSS Inc., Chicago, IL, USA). All values are given as a mean ± S.D. Significance is reported at the 5% level. Whenever comparisons were made with a common control, the significance of differences was tested by analysis of variance followed by Dunnett’s modification of the t-test.

## Data Availability

Not applicable.

## Supplementary Materials

The following are available online at https://www.mdpi.com/1422-0067/22/6/2902/s1, Supplementary Figure S1. Confocal microscopy detection of EEA-1 (red) and CyPA (green) in H9c2 cells cultured under hypoxic conditions and incubated with Ivabradine, Chloroquine and a combination of both. Merged panels show co-localization in yellow. *n* = 3.
