# Supplementary material for: Ivabradine Induces Cardiac Protection against Myocardial Infarction by Preventing Cyclophilin-A Secretion in Pigs under Coronary Ischemia/Reperfusion
Source: Int J Mol Sci. 2021 Mar 12;22(6):2902. doi: 10.3390/ijms22062902 (PMC8001911; doi:10.3390/ijms22062902)
Supplement: Supplementary file 1 [file ijms-22-02902-s001.pdf]

International Journal of
Molecular Sciences

MDPI

Supplementary Material

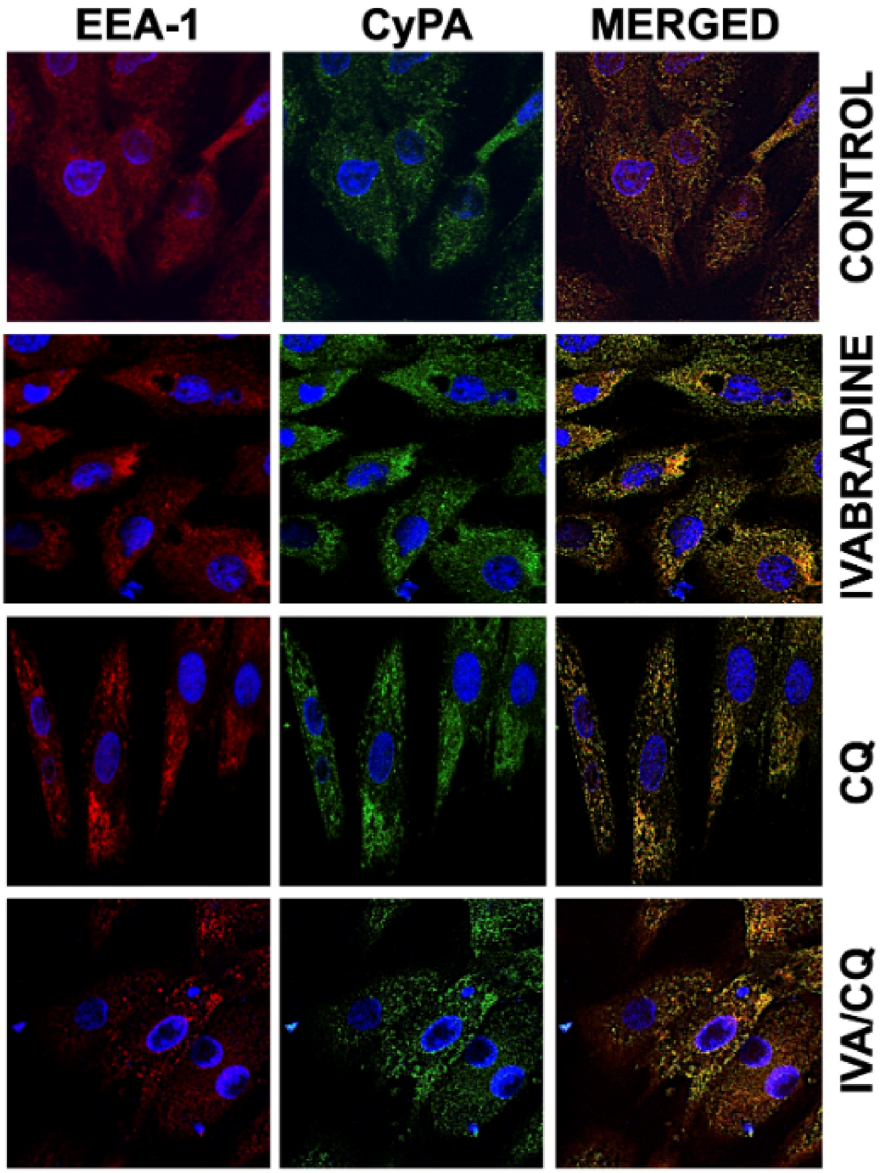

Figure 1. Confocal microscopy detection of EEA-1 (red) and CyPA (green) in H9c2 cells cultured under hypoxic conditions and incubated with Ivabradine, Chloroquine and a combination of both. Merged panels show co-localization in yellow. *n* = 3.
